# Oxytocin in infants with Prader-Willi syndrome to improve dysphagia and disease trajectory

**DOI:** 10.1186/s13023-026-04214-8

**Published:** 2026-02-04

**Authors:** Maithe Tauber, Gwenaelle Diene, Pascale Fichaux-Bourin, Graziella Pinto, Iva Gueorguieva, Marc Nicolino, Rachel Reynaud, Delphine Bernoux, Veronique Beauloye, Elin Malek Abrahimians, Cordula Kiewert, Pierre Payoux, Sophie Cabal, Catherine Molinas, Melanie Glattard, Sylvie Viaux-Savelon, Antoine Guedeney, David Cohen, Catherine Arnaud, Marion Valette

**Affiliations:** 1 Centre de Référence Maladies Rares PRADORT (syndrome de PRADer-Willi et autres Obésités Rares avec Troubles du comportement alimentaire), Hôpital des Enfants, CHU Toulouse, Université Toulouse III, Toulouse, France; 2 Institut Toulousain des Maladies Infectieuses et Inflammatoires (Infinity) INSERM UMR1291-CNRS UMR5051-Université Toulouse III, Toulouse, France; 3 Assistance Publique-Hôpitaux de Paris, Service d’Endocrinologie, Gynécologie et Diabétologie Pédiatrique, Hôpital Necker-Enfants Malades, Paris, France; 4 Département de Pédiatrie, CHU de Lille, Centre Intégré d’Obésité, Lille, France; 5 Service d’Endocrinologie Pédiatrique et Pédiatrie Générale, Hospices Civils de Lyon, Hôpital Femme Mère Enfant, 59 Boulevard Pinel, Bron, France; 6 Assistance-Publique des Hôpitaux de Marseille (APHM), Service Pédiatrique Multidisciplinaire - La Timone Hôpital des Enfants, 264 rue Saint Pierre, Marseille, France; 7 Zeepreventorium VZW, Koninklijke Baan 5, De Haan, Belgium; 8 Service de Pédiatrie Spécialisée Cliniques Universitaires Saint-Luc, Brussels, Belgium; 9 Division of Pediatric Endocrinology and Diabetes, University Hospital Essen, University of Duisburg, Essen, Germany; 10 Toulouse NeuroImaging Center (ToNIC), Université de Toulouse, Inserm, UPS, Toulouse, France; 11 Service de Psychiatrie de l’Enfant et de l’Adolescent, Hôpital des Enfants, Toulouse, France; 12 Hospices Civils de Lyon, Lyon, France; 13 Paris Cité Université, Paris, France, et Réseau Périnatal Occitan, Toulouse, France; 14 Institut des Pathologies du Développement de l’Enfant et de l’Adolescent (IDEAL), Hôpital Pitié-Salpêtrière, Assistance Publique-Hôpitaux de Paris, Sorbonne Université, Paris, France; 15 Unité de Soutien Méthodologique à la Recherche (USMR), CHU Toulouse, Toulouse, France

## Abstract

**Objective:**

Prader-Willi syndrome (PWS) is a genetic neurodevelopmental disorder with a characteristic trajectory. Infants display hypotonia, poor social and feeding skills, and high risk of choking, which have been shown to improve after oxytocin (OT) treatment. Our aim is to demonstrate the efficacy of intranasal OT treatment administered in the postnatal critical period on infant feeding skills and document its long-term effects.

**Methods:**

We enrolled 52 infants with PWS (median age 2.2 months) in a European double-blind randomized placebo-controlled study. Infants were randomly assigned in a 1:1 ratio to either 4 IU/day of OT or placebo for a 4-week evaluation of efficacy. A second randomization in each group was performed. Infants in the placebo group were randomized into 4 weeks of OT followed by 4 weeks of placebo or 8 consecutive weeks of OT. Those in the OT group were randomized into additional 4 weeks of OT followed by 4 weeks of placebo or 8 consecutive weeks of placebo. Infants were followed-up to 26 weeks from baseline. Feeding skills were evaluated using Neonatal Oral-Motor Scale (NOMAS) as primary endpoint and videofluoroscopy of swallowing study (VFSS) as key secondary endpoint. Subsequently, 40 infants included in France (OT-exposed cohort) participated in a new study to document long term safety of OT treatment and to compare them with an unexposed cohort (*n* = 24) at about 3 years of age. Prevalence and severity of comorbidities of the disorder were compared between the two cohorts.

**Results:**

OT was well tolerated. At 4 weeks, NOMAS normalization rates were similar between OT and placebo, but OT yielded a higher VFSS responder rate (53.3% vs 16.7%, *p* = 0.05) and a greater reduction in VFSS total score (LS mean difference −1.55; 95% CI −2.9 to −0.2; *p* = 0.03). At 3 years, the OT-exposed cohort demonstrated consistently better motor, adaptive and behavioral outcomes than unexposed controls.

**Conclusions:**

The primary endpoint did not show significant difference between OT and placebo groups. However, using VFSS we showed for the first time a positive effect of 4 weeks intranasal OT treatment on swallowing. We also document long-term effect on disease trajectory, with less severe comorbidities.

**Supplementary Information:**

The online version contains supplementary material available at 10.1186/s13023-026-04214-8.

## Background

Prader-Willi syndrome (PWS) is a rare neurodevelopmental genetic condition caused by the loss of expression of paternally inherited imprinted genes due to deletion of chromosome 15q11-q13, uniparental disomy, or imprinting defect [1, 2]. Genetic diagnosis is now made in the first months of life in industrialized countries, enabling early intervention and management with family support. PWS is characterized by impaired developmental trajectories, including nutritional, endocrine/metabolic, and behavioral disturbances related to hypothalamic dysfunction [3]. Neonates and infants display marked hypotonia, feeding difficulties with poor suck, and swallowing deficits requiring nasogastric tube feeding to ensure normal weight gain and prevent risk of choking and life-threatening complications [4]. The role of the PWS chromosomal region—particularly the maternal imprinted *MAGEL2* and *SNORD116* genes [5–7]—is crucial to the development and function of oxytocin (OT)-producing neurons. Indeed, preclinical data from *Magel2* gene-inactivated mouse model have shown rescue of suckling in pups and normalization of learning, memory and social cognition in adult mice following a single OT injection before the first 5 hours of life [5, 8]. In our previous phase II clinical study, infants with PWS under 5 months who received 7 days of intranasal OT showed improvements in sucking and swallowing, social withdrawal, and mother-infant interactions [9, 10]. These clinical changes were correlated with increased connectivity of the right superior orbitofrontal cortex. Intranasal OT was well tolerated [9].

We next implemented a European multicenter placebo-controlled study (OTBB3) to demonstrate the effects of intranasal OT in infants. The treated children followed in expert centers in France were then invited to participate in the OTBB3-follow-up study (OTBB3-FUP) to assess long-term safety up to 4 years and to compare them with an unexposed cohort of children with PWS also followed in the expert centers. We report here the results of the OTBB3 study and the interim analysis of OTBB3-FUP at about 3 years.

## Methods

### Study oversight

OTBB3 (Trial registration: Clinical trial, NCT04283578, registered 19 February 2020, https://clinicaltrials.gov/study/NCT04283578?cond=NCT04283578&rank=1) is a phase III, European, multicenter, randomized, double-blinded placebo-controlled superiority clinical study conducted in three European countries. OTBB3-FUP (Trial registration: Clinical trial, NCT05032326, registered 26 August 2021, https://clinicaltrials.gov/study/NCT05032326?cond=prader-willi&rank=8) is a prospective cohort study conducted in 11 French centers. All parents gave written informed consent. Studies were performed according to the principles of the Declaration of Helsinki and regulatory requirements. Both studies are investigator-led. The authors assume responsibility for the accuracy and completeness of the data and analyses and the fidelity of the study to the protocol. Additional details on the protocols and statistical analysis plans can be obtained by request.

### Study procedures

#### OTBB3 design

**Fig. 1:**
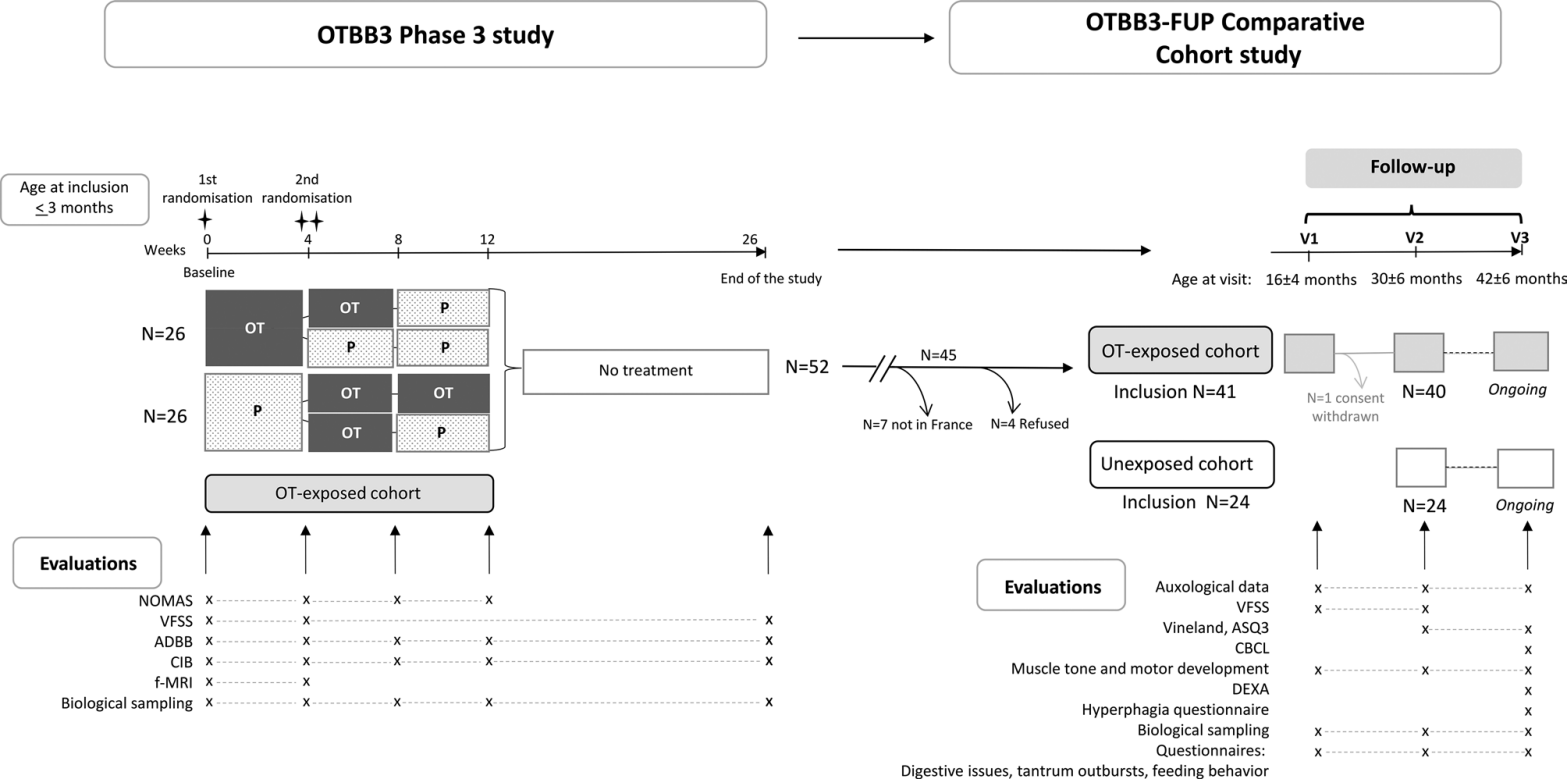
OTBB3 and OTBB3-FUP designs. The designs of the two studies are shown with the evaluations and their timelines. Among 52 infants included in the OTBB3 study 45 were included in France, 41 were included in OTBB3 FUP study and 1 family withdrew consent after visit 1 (V1). At visit 2 (V2) around 3 years old, 40 OT-exposed children were compared to 24 unexposed children. These 2 cohorts will be followed up to 4 years old (V3)

In each center, infants diagnosed with PWS and under 3 months old were enrolled and randomly assigned in a 1:1 ratio to either 4 IU/day of OT or placebo for a 4-week evaluation of efficacy. For ethical reasons, a second randomization in both OT and placebo groups ensured that all infants received at least 4 weeks of OT (Fig. 1). Infants in the placebo group were randomized into 4 weeks of OT followed by 4 weeks of placebo or 8 consecutive weeks of OT treatment. Those in the OT group were randomized into additional 4 weeks of OT followed by 4 weeks of placebo or additional 8 weeks of placebo. The whole treatment period was stopped after 12 weeks from baseline. All infants were subsequently followed up to 26 weeks from baseline.

Investigational drug: OT was administered as a nasal spray, supplied by OT4B (Toulouse, France) and specially designed for appropriate administration in neonates. It delivers 2 IU of oxytocin per spray, and administration consists of 2 sprays per day (one spray in each nostril, preferably). The device is specially adapted for infants. During the OTBB3 study, the product was administered in the morning by parents or health care providers.

#### OTBB3-FUP design

Children enrolled in OTBB3 and followed in the French centers (OT-exposed group) had a first visit at 16 ± 4 months (V1) a second visit at 30 ± 6 months (V2) and a third visit at 42 ± 6 months (V3). The unexposed cohort included children who could not participate in OTBB3 study because they were older than 3 months before the study commenced or at their first visit in the expert centers. These unexposed children were included at 30 ± 6 months (V2) (Fig. 1). The two cohorts were followed in the French centers regarding the same recommendations of care, thus allowing comparisons between the two cohorts at each visit.

#### OTBB3 endpoints

Endpoints were the same used in our previous phase II study [9]. We used the Neonatal Oral-Motor Assessment Scale (NOMAS) with the same scoring as previously published [9]. In this current multicenter study scoring was performed on videos of feeding and not at bedside as in our previous monocentric phase II study. Total score varies between 8 and 28, with a score ≤10 defining a near-normal sucking pattern. The VFSS was scored with a grid of 9 relevant items used in our previous study and in routine practice in our center (supplementary Table 1). VFSS was normal if the score was 11, and the maximum abnormal score was 29. In this current study NOMAS and VFSS were centrally scored in a blinded manner. For the primary endpoint videos of feeding were scored by 2 independent trained experts.

Feeding efficiency was evaluated by the proficiency score defined as the volume of milk taken in the first 5 minutes of feeding expressed as % of the proposed milk volume (normal score ≥40%).

*Primary endpoint* was the proportion of neonates/infants who achieved normal or subnormal NOMAS scores (≤10) at week 4.

*Secondary endpoint* was the proportion of infants with abnormal VFSS scores at baseline on at least one of the two items associated with high choking risk: pharyngeal propulsion and airways protection, and who reached normal scores at week 4 considered as responders. Changes in VFSS total score were also evaluated in the 2 groups at week 4 (see exploratory endpoints).

*Other secondary endpoints at week 4* included:

- Social skills evaluated on the videos of feeding using the Alarm Distress Baby Scale (ADBB, normal score < 5) for social withdrawal and the Coding Interactive Behavior (CIB) scale for mother -infant interactions comprising 7 sub scores (parental sensitivity, parental intrusiveness, dyadic reciprocity, child withdrawal, dyadic joint negative state, child social engagement and child state).
- Changes in total circulating ghrelin, acylated ghrelin (AG) and unacylated ghrelin (UAG) levels quantified using an ELISA kit on fasting plasma samples.

*Exploratory endpoints* included changes in VFSS total score as described above in the VFSS section, brain connectivity measured using resting-state functional MRI (rs-fMRI) [9], and feeding and social skills at weeks 8, 12 and 26. NOMAS was not scored at week 26 as it has not be developed and used for infants older than 6 months.

*Safety measures*

At each visit, vital signs (blood pressure and heart rate), electrocardiogram before and within 30 minutes after each treatment administration, safety biological parameters, and urine density were assessed.

### Endpoints of the OTBB3-FUP study

*Primary endpoint* was long-term safety up to 4 years in children of the exposed cohort, including assessment of vital signs, adverse events (AE) and secondary endpoints were the comparison of the comorbidities between the two cohorts at V2 and V3. This comprised clinical evaluations of hypotonia, growth (height, weight, BMI), motor tone and motor milestones (age at sitting, crawling, walking and running), orthopedic issues (presence of kyphosis and scoliosis), ocular problems assessing the occurrence and treatment of strabismus, myopia, astigmatism, hypermetropia, dysautonomia features (dysphagia evaluated by VFSS and other gastrointestinal symptoms assessed by the digestive disorder’s questionnaire filled by the parents during physician interview, type of stools evaluated by Bristol scale and apnea hypopnea index calculated on polysomnography), behavior measured by the Vineland Adaptive Behavior Scale, Second Edition (VABS-II) and the Ages and Stages Questionnaires, Third Edition (ASQ3), and questionnaires assessing tantrum outbursts [11], eating behavior [12] filled by parents and nutritional phase status evaluated by physicians [4].

### Statistical analyses

#### OTBB3 study

Primary (NOMAS responder) and key secondary (VFSS responder) endpoints were analyzed in the full analysis set (all randomized patients) by exact conditional logistic regression, including treatment and stratified by pooled site, yielding exact Odds Ratios (ORs) with 95% Confidence Interval (95%CI) and two-sided *p* values (α = 0.05).

Other continuous secondary endpoints were evaluated by Analysis of Covariance (ANCOVA) or mixed models for repeated measures (MMRM).

Type I error was controlled at the two-sided 0.05 level using a fixed-sequence testing procedure for the primary and ordered key secondary endpoints. As the primary endpoint did not reach statistical significance, all key secondary and secondary endpoints are reported with nominal p-values, which should thus be interpreted with caution.

To take into account differences in family environment, we assessed social status using the Barratt Simplified Measure of Social Status (BSMSS) and social support of the family using the Social Provision Scale (SPS). Primary and secondary endpoints at week 4 were then analyzed by high, medium or low categories of BSMSS and SPS.

#### OTBB3-FUP study

Binary endpoints were analyzed using Fisher’s exact test, and continuous endpoints using the Mann–Whitney test. All reported p-values are nominal (i.e., unadjusted for multiplicity).

All analyses were conducted using SAS® version 9.4.

## Results

### OTBB3 population characteristics

Fifty-two infants were included in Europe with a mean age of 2.2 months. For the first 4 weeks of the study, the OT and placebo groups comprised 26 infants each with no substantial differences regarding age, genetic subtype, birth, and feeding disorders (Table 1).

**Table 1:** Characteristics of the OTBB3 and OTBB3-FUP populations

Characteristics of the OTBB3 population at inclusion			P
	OT group	Placebogroup	
	N = 26	N = 26	
Female sex – n (%)	12 (46%)	12 (46%)	NS
Genetic diagnosis – n (%)			
Deletion	16 (62%)	16 (62%)	
UPD	9 (34%)	9 (34%)	
Abnormal methylation profile	1 (4%)	1 (4%)	
**Birth data:**			
Gestational age – weeks of amenorrhea	38.1 (32.6;41.3)	39.7 (33.1;42.0)	
Preterm birth (< 37 weeks) – n (%)	8 (31%)	7 (27%)	
Cesarean delivery – n (%)	18 (69%)	12 (46%)	
Weight – SDS	1.8 (−6.6;0.4)	−1.4 (−5.7;0)	
Length - SDS	−1.5 (−5.3;1.9)	−0.6 (−5.2;4.2)	
**Data at baseline:**			
Age – months	2.2 (0.4;4.0)	2.2 (0.7;4.7)	
Weight – SDS	−2.6 (−4.1;–0.8)	−2.5 (−6.0;–0.4)	
Length – SDS	−2.0 (−4.4;0.2)	−1.5 (−6.0;1.0)	
BMI – SDS	−2.2 (−3.7;–02)	−2.0 (−3.7;–0.3)	
Tube feeding – n (%)	9 (35%)	14 (54%)	
**Characteristics of the OTBB3-FUP population at V2**			
	**OT-exposed cohort**	**Unexposed cohort**	**P**
	**N = 40**	**N = 24**	
Female sex – n (%)	18 (45%)	11 (46%)	NS
Genetic diagnosis – n (%)			NS
Deletion	24 (60%)	11 (46%)	
No deletion (UPD, Imprinting defect)	14 (35%)	12 (50%)	
Abnormal methylation profile	2 (5%)	1 (4%)	
Age (months)	32 (27;38)	36 (26;42)	< 0.001
Weight -SDS	−0.49 (−2.5;1.8)	−0.30 (−2.6;4.3)	0.02
Height - SDS	−1.03 (−4.0;1.0)	−0.80 (−3.8;1.0)	0.05
BMI - SDS	0.06 (−1.7;4.3)	0.64 (−1.7;5.6)	NS
Use of GH medication – n (%)	38 (95%)	24 (100%)	NS
Age at start GH medication - yrs	0.8 (0.2;3.4)	0.8 (0.1;2.8)	NS

### OTBB3 results at week 4

#### Oral skills and dysphagia

*NOMAS:* Percentage of responders using NOMAS (score ≤10): no difference was observed between OT and placebo groups, with five responders (19%) in each group.

**Fig. 2:**
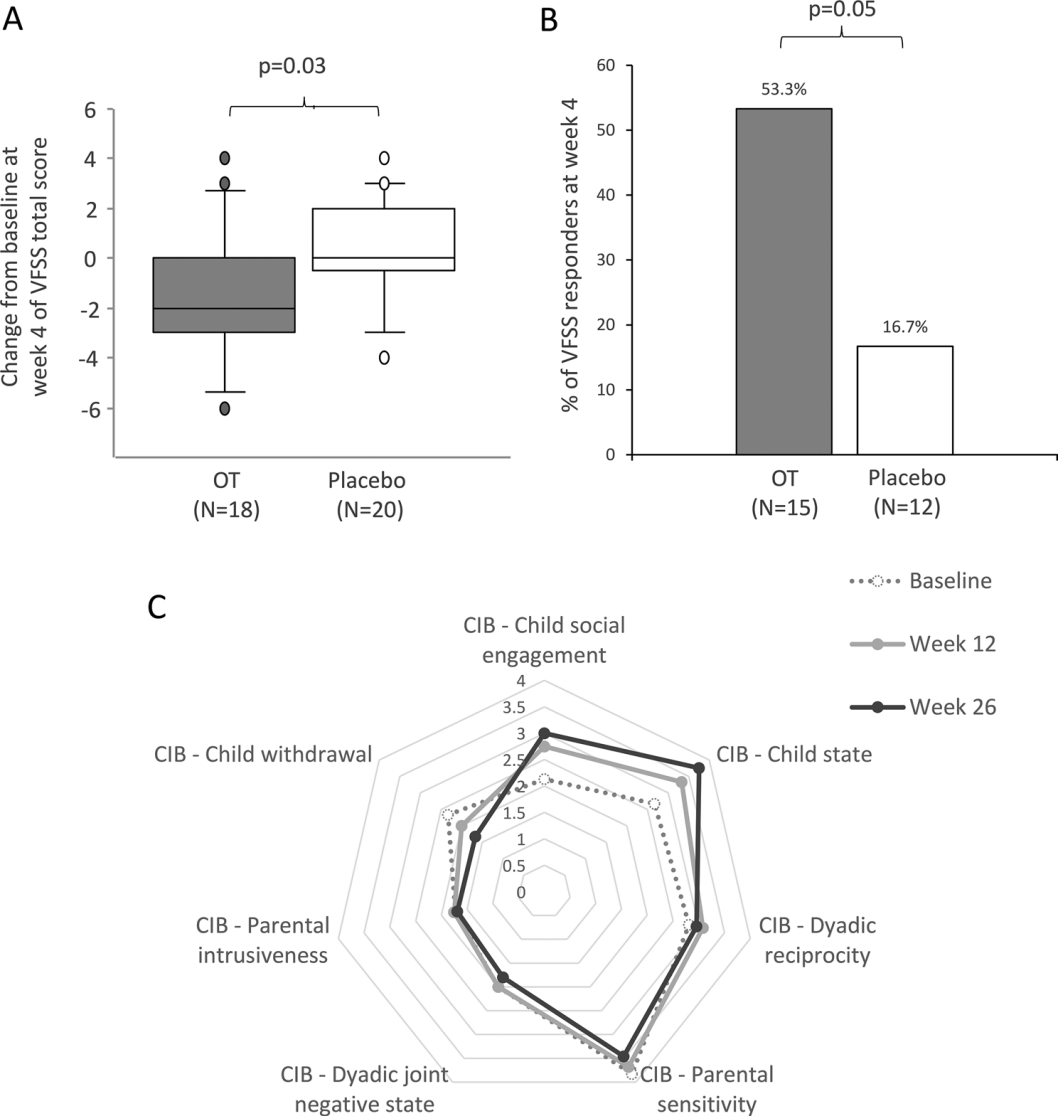
Analyses of videofluoroscopic swallowing study (VFSS) and social skills in the OTBB3 study. The VFSS grid includes 11 items. All normal evaluations are scored 1 and abnormal assessment may be scored 2 to 5. The total score is calculated by adding all points. The minimum score is 11 and the maximum score is 29 [9]. (**A**) proportion of infants with pharyngeal residuals or penetrations on VFSS at inclusion who were normal for these two items after 4 weeks of treatment in OT group (grey box) vs placebo group (white box); p-value from a two-sided exact cochran-mantel-hansel (CMH) test. (**B**) change from baseline of VFSS total score at week 4 in OT group (grey box) vs placebo group (white box); p-value from an analysis of covariance (ANCOVA). (**C**) median CIB- Sub scores of the whole group of infants at baseline (dotted lines), week 12 (gray lines) and week 26 (black lines). This drawing clearly shows that child state, child social engagement increased with time with concomitant decrease of child withdrawal

*VFSS:* VFSS was scored in 42 infants at baseline and in 38 infants both at baseline and week 4 (VFSS at week 4 was not performed for 2 infants and made but not interpretable for 2). At baseline, 39 infants among 42 had abnormal VFSS total score (93%). When analyzed by percentage of responders, at baseline, 27/38 infants with VFSS scored had abnormal sub scores (71%) in pharyngeal propulsion (15 in OT group and 12 in placebo group) and, among them, 8 (4 in each group) also had abnormal sub scores for airway protection. Eight among fifteen (53.3%) in OT group vs 2/12 (16.7%) in placebo group normalized these two sub scores (*p* = 0.05) with OR of 7.05 (95% CI: 0.98–87.13) (Fig. 2A).

Analyses on the 38 VFSS performed both at baseline and week 4, showed a difference between the change in VFSS total score from baseline in OT (*N* = 18) vs placebo group (*N* = 20) (difference LS means −1.55, 95 CI (−2.9 to −0.2), *p* = 0.03) (Fig. 2B). Sixty-one percent (11/18) of infants in OT group improved vs 25% (5/20) in placebo group (*p* = 0.06) with 7/18 OT-treated children (39%) improved by 3 points or more vs 2/20 (10%) in placebo group.

*Feeding efficiency:* no difference was found in the proficiency score between OT and placebo groups.

#### Social skills

No difference was found in ADBB, CIB between OT and placebo groups. When analyzed by family social support categories, ADBB score change from baseline to week 4 was greater (i.e., improved) in OT group vs placebo in infants with high family SPS score [−2.0 (*n* = 5) vs 1.0 (*n* = 7), difference LS Means −3.0, 95 CI (−6.1 to −0.1), *p* = 0.04]. When analyzed by family social status categories, CIB dyadic reciprocity sub score change from baseline to week 4 was better in infants with low BSMSS score [0.25 (*n* = 6) vs −0.15 (*n* = 6), difference LS Means 0.1, 95 CI (0.1 to 1.6), *p* = 0.03].

#### Ghrelin levels

No between-group difference was observed for changes in circulating AG, UAG or total ghrelin.

**Fig. 3:**
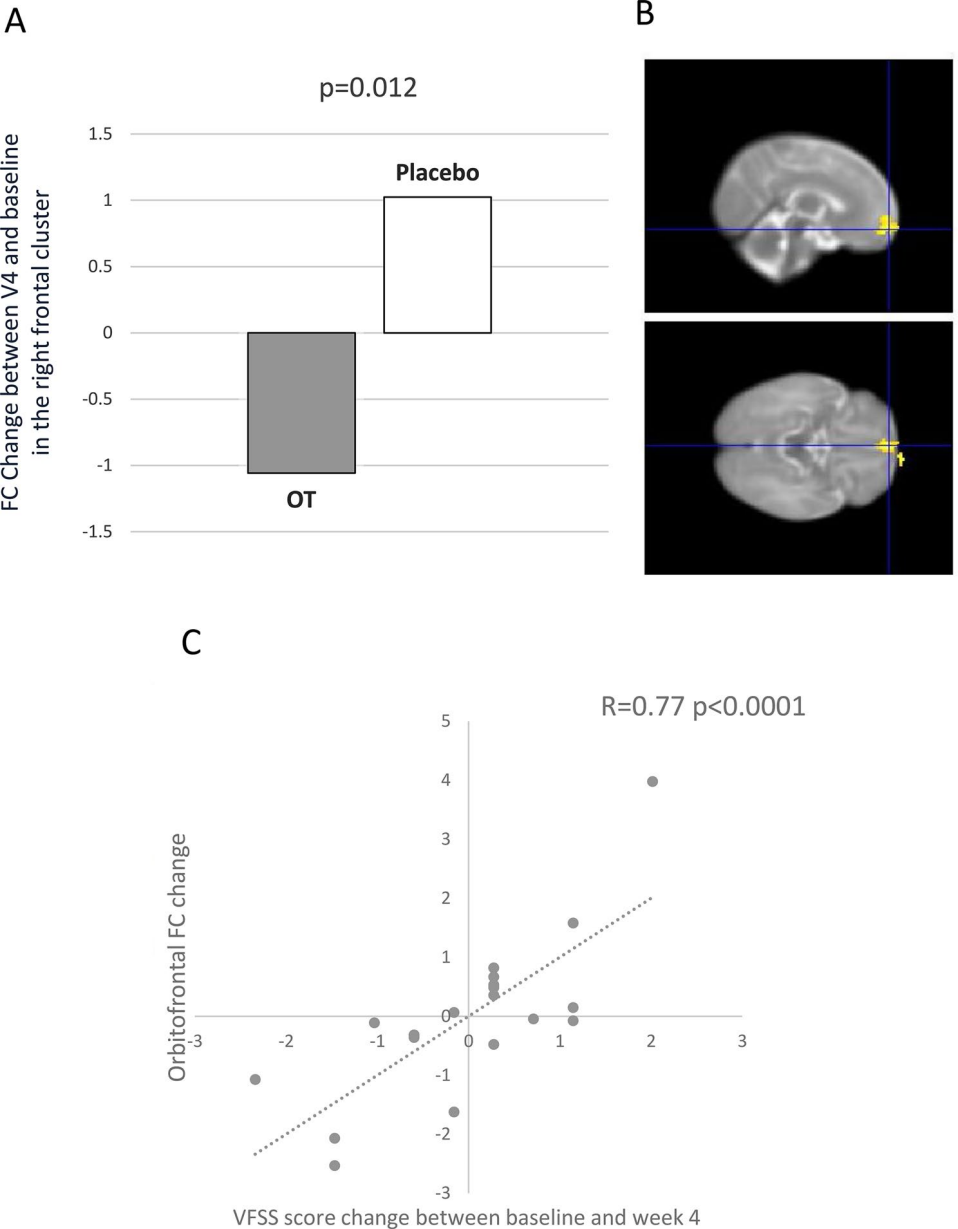
Rs-fMRI results in OTBB3 study. (**A**) Bar chart shows the functional connectivity (FC) change from baseline to week 4 in OT group (grey bar) and placebo group (white bar) in the right frontal lobe. (**B**) sagittal (up) and axial (bottom) views show the orbitofrontal cortex region which change in functional connectivity (FC) from baseline to week 4 is positively correlated with VFSS score change in the whole population. (**C**) correlation between orbitofrontal region FC change and VFSS total score change from baseline to week 4 in the whole population (*n* = 19)

*rs-fMRI:* 25 rs-fMRI were considered as high quality to be analyzed both at baseline and week 4. In these 25 rs-fMRI, we found a difference in the change of functional connectivity in the right frontal lobe (*p* = 0.01), which decreased in OT-treated group (*n* = 11) and increased in placebo group (*n* = 14) (Figs. 3A, B). In the whole population we found a positive correlation between the change in connectivity in the orbitofrontal cortex region and the change in VFSS score from baseline to week 4 (*n* = 19) (Fig. 3C).

### OTBB3 results at week 12 and week 26

Feeding skills continued to improve over time in all groups, median NOMAS score decreased from 17 at baseline to 12 at week 12, with 44% of infants reaching normal scores, median VFSS total score decreased from 15 at baseline to 13 at week 26, with 51% reaching normal scores. The group that started OT first achieved lower median VFSS total score at week 26 compared to the delayed start group (12.5 vs 14.0, *p* = 0.002), and among this OT-first group, the group that received 8 vs 4 weeks of OT achieved a lower medium VFSS total score at week 26 (LS Means −3.23 95 CI (−5.1; −1.3) vs −1.16 95 CI (−3.2;0.9), *p* = 0.002). Social skills also continued to improve over time with 51% of infants achieving normal ADBB scores at week 26. All CIB sub scores showed improvement (Fig. 2C).

#### Safety

**From baseline to week 4**, 64 adverse events (AE) were reported in 32/52 children (62%) with no difference between OT (27AE) vs placebo (37 AE) group. Treatment emergent AE (TEAE (in 13 patients of OT group vs 19 of placebo group) are described in Supplementary Table 2. Prevalence and severity summary of AE and TEAE are shown in Supplementary Table 3. No pulmonary infection was reported and bronchiolitis occurred in two infants in the Placebo group vs none in the OT group.

**From baseline to week 26**, TEAE (in 54 patients in 4 weeks OT group vs 63 in 8 weeks OT group) are described in Supplementary Table 4. No difference was observed in relation to the duration of OT treatment. Most TEAE were of mild or moderate intensity and unrelated to treatment as shown in Supplementary Table 5.

During the whole study, bronchiolitis occurred in 12 infants (23%) corresponding to a normal incidence at this age.

### OTBB3-FUP results

#### OTBB3-FUP population characteristics

Forty-one among the 45 French children included in the OTBB3 study (4 weeks of OT treatment, *n* = 21 and 8 weeks, *n* = 20), were enrolled in OTBB3-FUP study along with 24 unexposed children with PWS. Parents of one child of the OT-exposed cohort withdrew their consent for personal reason after visit V1. Sixty-four children of both cohorts were therefore analyzed at V2 (planned interim analysis) at about 3 years. Table 1 describes the characteristics of these children. All children but two (of the OT-exposed cohort whose parents refused to start treatment at the time of analysis) were treated with growth hormone (GH).

#### Safety

Serious AE unrelated to treatment were reported for eight (20%) OT-exposed children and three (13%) unexposed children, mainly infections (e.g., gastroenteritis, influenza, pneumonia, febrile convulsion and asthma), consistent with the young age of the children.

#### Comparison between the two cohorts at visit 2, at 30 ± 6 months

Age was lower in OT-exposed children (32 months, from 27 to 38) compared to unexposed children (36 months, from 26 to 42), (*p* < 0.001). Height and weight expressed in standard deviation score (SDS) were lower in OT-exposed children (*p* = 0.05 and *p* = 0.02, respectively) while BMI was not different (Table 1).

**Fig. 4:**
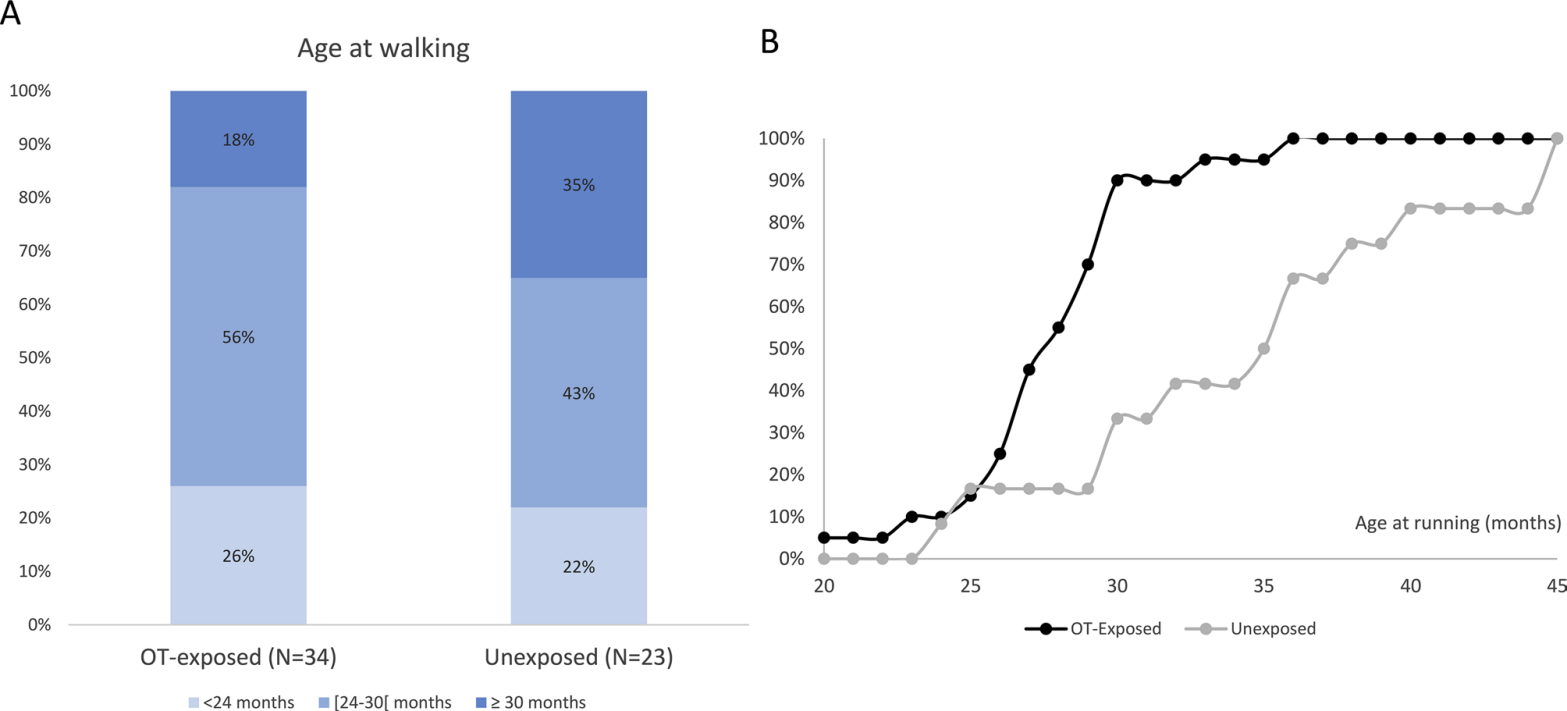
Timing of walking and running acquisition in the two cohorts of OTBB3-FUP study. For these analyses we gathered all data available from visits V2 and V3 at the time of interim analysis. (**A**) distribution of categories of age at walking in the OT-exposed group (*N* = 34) and the unexposed group (*N* = 23). (**B**) cumulative graphs for the age at running acquisition in the OT-exposed group (*N* = 20, dark grey) and the unexposed group (*N* = 11, light grey)

Other results are shown in Table 2 and Fig. 4.

- **Muscle tone and psychomotor development:** There were many differences, in favor of better development of the OT-exposed children, including no (*n* = 3 vs 0) or less severe hypotonia than usually observed in PWS (*n* = 11 vs 3) (37 vs 14%, *p* = 0.06), no kyphosis vs 25% (*p* = 0.002), less frequent severe walking delay (Fig 4A), lower age at running (Fig 4B) with a median age of running acquisition of 28.5 months (*N* = 20 in OT-exposed group) vs 38.5 months (*N* = 12 in unexposed group) (*p* = 0.02). Fewer children in the OT-exposed cohort had low scores (≤-2 SD) for fine motor skills on ASQ-3 (33% vs 64%, *p* = 0.02) and gross and global motor skills on VABS-II (50% vs 83%, *p* = 0.002 and 47% vs 78%, *p* = 0.01, respectively). Ocular disorders occurred in 48% of OT-exposed children vs 67%, with strabismus in 35% of OT-exposed children vs 46%.
- **Dysautonomia:** OT-exposed children displayed fewer features of dysautonomia including lower apnea-hypopnea index before the initiation of GH treatment (3.5 vs 7.0/hr, *p* = 0.45), more normal/subnormal esophageal motility VFSS sub score (64% vs 35%, *p* = 0.06), less frequent bad breath (16% vs 37%, *p* = 0.05), and less frequent large quantities of stools (47% vs 83%, *p* = 0.02) evaluated by the digestive disorder’s questionnaire.
- **Behavior**: albeit median scores for the 4 dimensions of the VABS-II are not different between the 2 cohorts, analyses by categories showed that OT-exposed children have fewer low adaptive levels for socialization (26% vs 48%, *p* = 0.09), fewer low composite adaptive behavior score (53% vs 74%, *p* = 0.1). In addition, OT-exposed children had significantly less frequent temper outbursts (*p* = 0.01), less hetero- (*p* = 0.009) and auto-aggressivity (*p* = 0.06) evaluated by families.

**Table 2:** Comparisons of comorbidities: muscle and motor outcome, behavior, and dysautonomia features between the OT-exposed and unexposed cohorts in the OTBB3-FUP study at visit 2, at 30 ± 6 months

	OT-exposed	Unexposed	P
	N = 40	N = 24	
**Muscle tone and motor development**			
Hypotonia – n (%)	35/39 (90%)	24 (100%)	0.06
No hypotonia or less severe than usual for PWS	14/38^*^ (37%)	3 (14%)	
Usual for PWS or more severe than usual for PWS	24/38^*^ (63%)	19 (86%)	
Scoliosis – n (%)	9 (23%)	8 (33%)	0.39
If yes, age at diagnosis (Min;Max) - months	16 (6;32)	13 (6;38)	
Kyphosis – n (%)	0/39 (0%)	6 (25%)	0.002
If yes, age at diagnosis (Min;Max) - months	-	30 (13;37)	
ASQ3 Fine motor score (Min;Max) - SN	22.5 (0;55)	10 (0;55) *N* = 22	0.08
Score ≤ −2 SD of the age category – n (%)	13 (33%)	14 (64%) *N* = 22	0.02
VABS-II: Motor skills domain (Min;Max) – SN	73 (22;103) *N* = 38	61 (24;124) *N* = 23	0.08
Low score of the age category:			
Motor skills domain – n(%)	19 (50%)	19 (83%)	0.01
Gross motor – n(%)	18 (47%)	18 (78%)	0.02
Strabismus – n (%)	14 (35%)	11 (46%)	0.39
**Behavior**			
VABS-II scores:	*N* = 38	*N* = 23	
Communication domain (Min;Max) - SN	79 (42;107)	79 (45;103)	0.61
Daily living skills domain (Min;Max) - SN	76 (35–160)	71 (31;137)	0.6
Socialization domain (Min;Max) - SN	75 (41;151)	71 (48;120)	0.2
Adaptive behavior (Min;Max) - CS	68 (24;134)	64 (30;127)	0.23
% of children with temper outburst in the last month – n (%)	27 (68%)	16 (67%)	0.63
If Yes, number of outbursts within the last month:			
1–3/month – n (%)	14 (52%)	3 (19%)	
1–3/week – n (%)	8 (30%)	4 (25%)	0.01
≥1/day – n (%)	5 (18%)	9 (56%)	
Hetero-aggressivity – n (%)	1 (3%)	6 (25%)	0.009
Auto-aggressivity – n (%)	1 (3%)	4 (17%)	0.06
**Dysautonomia**			
VFSS Total score (Min;Max)	5 (1;12) (*N* = 28)	5 (3;9) (*N* = 16)	0.85
% normal/subnormal VFSS dysmotility subscore	62%	35%	0.08
Gastrointestinal issues:	(*N* = 38)		
Large quantities of stool			
Never – n (%)	2 (53%)	4 (17%)	0.02
Rare – no (%)	13 (34%)	15 (63%)	
Common – n (%)	3 (8%)	4 (17%)	
Very common – n (%)	2 (5%)	1 (4%)	
Bad breath			
Never – n (%)	21 (55%)	10 (42%)	0.05
Rare – no (%)	11 (29%)	5 (21%)	
Common – n (%)	5 (13%)	6 (25%)	
Very common – n (%)	1 (3%)	3 (12%)	
Polysomnography before GH treatment collected in medical file **			
Age (Min;Max) –months	7.5 (1;14) (*N* = 37)	5.8 (1;15.0) (*N* = 21)	0.2
AHI (Min;Max) –/hr	3.5 (0.6;32) (*N* = 32)	7.0 (0.4;38) (*N* = 19)	0.45
Time spent with SAO_2_ < 90% (Min;Max) –min	0.12 (0;5) (*N* = 21)	1.0 (0;124) (*N* = 13)	0.1

SD: standard deviation; SN: standard note; CS: composite score. Results are expressed in median (Min-Max) or in n (%). Numbers are added when different for the sample size of the 2 cohorts

*1 missing data

**Polysomnography was performed before start of GH treatment and not at visit 2

## Discussion

These two studies document for the first time the effects of early intranasal OT treatment on neonates and infants with PWS and showed favorable short- and long-term safety profiles. We demonstrated an early effect of OT treatment on swallowing, evaluated by VFSS in infants, and a strong long-term motor effect and less severe condition around 3 years.

OTBB3 study did not reveal a difference in the percentage of responders after 4 weeks of OT treatment using NOMAS. This is possibly due to the observed wide intra- and inter-variability of infant NOMAS score obtained on centralized scoring on the feeding video. This variability may be related to the difficulties of having a video of feeding which represents the real feeding skills of the infants, likely due to the study team time constraints which we did not anticipate. Indeed, our previous study which was monocentric with complete hospitalization for the whole study duration facilitated the evaluation of feeding skills at bedside at the best moment. Interestingly using VFSS, we found a higher proportion of responders in the OT group (53.3% vs 16.7% in placebo group), with OR of 7.05 (95% C; 0.98–87.13) indicating a lower risk of choking and associated life-threatening complications. No severe pulmonary infections were reported during the whole duration of OTBB3 study and normal frequency of viral bronchiolitis were observed. In addition, VFSS total score improvement at week 4 was higher in OT group compared to placebo group. This improvement continued after cessation of the treatment period up to week 26, with more than 50% of infants achieving normal VFSS total score. This high percentage of normal VFSS contrasts with our observations in routine care and with published studies with abnormal VFSS reported in over 90% of patients with PWS at all ages [13–15]. We also found between-group differences (OT vs Placebo after 4 weeks) in social skills when family context, such as social support and social status, was considered, which highlight the interactions between OT treatment and social context. We speculate that parents with high social support meaning that they have higher family and/or friends support help them to cope with the burden of having a baby with PWS, so that they can interact more with their infant to reduce their social withdrawal. Again, social skills continued to improve up to 26 weeks with better scores compared to routine observations at the same age [9]. Furthermore, we confirmed that the orbitofrontal region is a target region for early OT treatment, as we previously reported [9]. We found highly clinically relevant that changes in orbitofrontal cortex region connectivity were correlated with changes in VFSS scores after 4 weeks of OT treatment. Conversely to our previous phase II study we did not find differences in ghrelin levels at week 4 between the 2 groups OT and Placebo which may be explained by differences on lower age at inclusion, longer duration of OT treatment and the wide variability of ghrelin levels in this population [16].

**Fig. 5:**
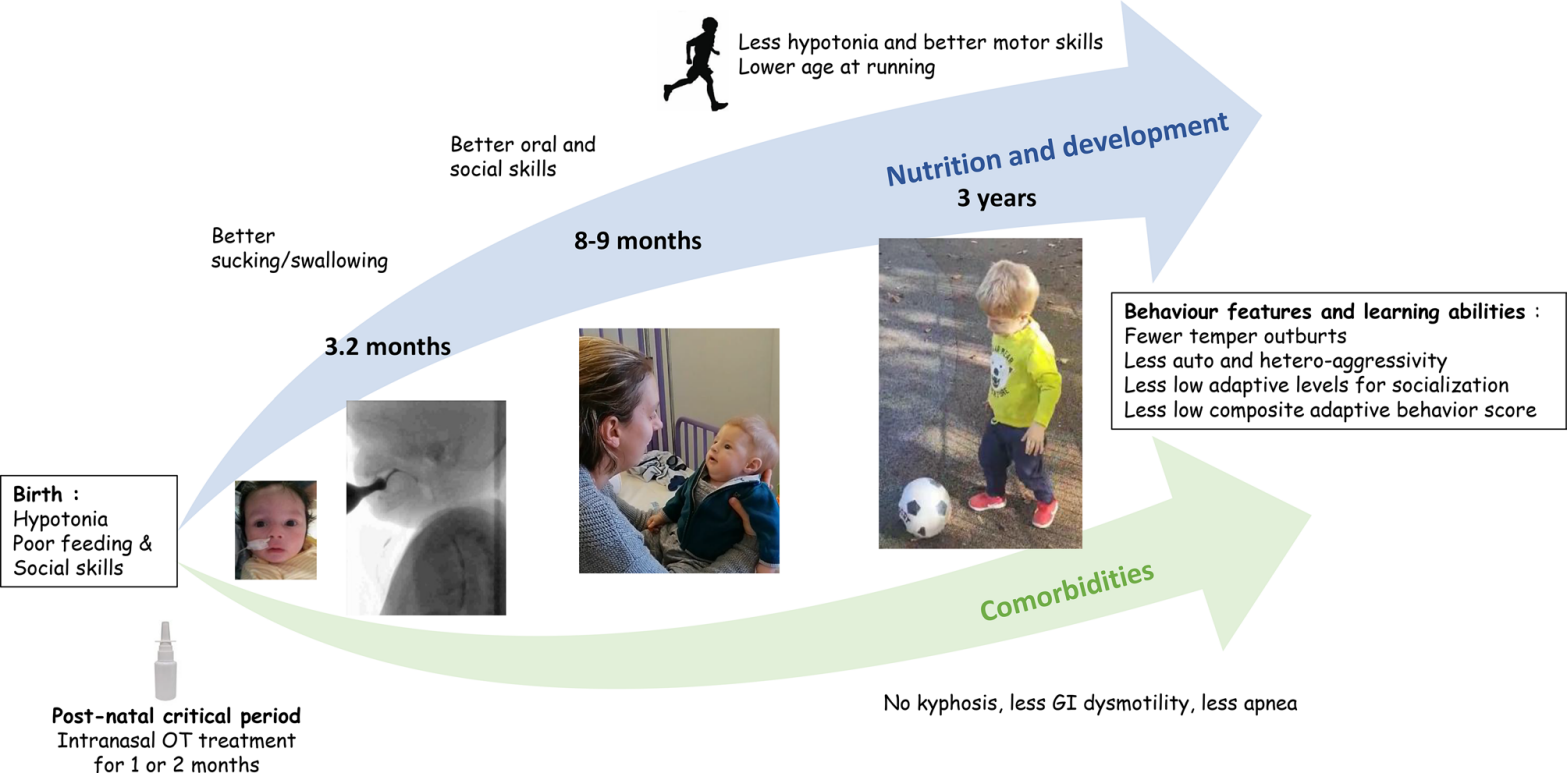
Short- and long-term effects of early intranasal OT treatment. The picture is based on our previous publication [9] to show all the effects of early OT treatment at 3 times points, after 4 weeks, 26 weeks of treatment and at 3 years of age, and the modifications in disease trajectory. The arrows show the trajectories on OT treatment including nutrition, development and comorbidities. Between the arrows major differences observed in OT-exposed cohort compared to unexposed cohort in behavioral features and learning abilities are listed

The OTBB3-FUP study demonstrated for the first time that OT-exposed children achieved better outcomes around 3 years regarding muscle tone and motor skills, dysautonomia, adaptive skills and behavior. Overall the results showed convergent signals indicating that early OT treatment is associated with a strong motor signature and change in disease trajectory with fewer OT-exposed children displaying a severe condition at 3 years. We propose a figure to summarize the short- and long-term effect of early OT treatment (Fig. 5). Swallowing improvement using VFSS appeared to be the first observed motor effect of early OT treatment, subsequently linked to broader enhancements in motor skills. Interestingly this has also been reported in Parkinson’s disease a neurodegenerative condition where dysphagia usually precedes motor dysfunction in patients [17]. Overall, the motor development of OT-exposed children surpassed that of their counterparts. This included all developmental motor milestones, particularly running acquisition, fine and gross motor skills, better muscle tone with no observed kyphosis, less strabismus, and better esophageal and GI motility. These early OT-exposed children not only run at an earlier age, but they do so with completely normal coordination, which is very rarely observed in PWS. They also display less severe walking delay with the exception of children who display pronounced hyperlaxity (20 to 30% in PWS) that prevents early walking acquisition. OT has been involved in thermogenesis regulation [18], which is closely linked to the tonic action of OT in skeletal muscles, similar to what occurs in the uterus, known as the “oxytonic effect” [19, 20]. Early OT treatment may correct the imbalance between central and peripheral OT systems observed in PWS, characterized by low hypothalamic secretion and elevated circulating OT levels [18]. Based on the data obtained in *Magel2* KO mice receiving OT treatment in the neonatal phase [8] which document an increase of mature OT secretion in the hypothalamic PVN and a normalization of the number of OT receptors in various brain regions, we speculate that OT treatment in infants with PWS also exerts a positive feed-back on OT neurons which increases hypothalamic OT secretion that in turn by a negative feed-back loop decreases circulating OT.

According to the framework of developmental cascades, motor improvement subsequently impacts adaptive skills and behavior [21]. Indeed, we observed better socialization and fewer temper outbursts at 3 years. Our results go in the same direction of those of the preclinical studies in *Magel2* gene-inactivated mouse model, which have shown improved sucking in pups and better outcomes in adults after early OT treatment [8] and confirm that the early postnatal stage is the unique critical period for oxytocin signaling to modulate brain development [22]. We did not observe difference in feeding behavior, indeed hyperphagia usually starts after 4 years in PWS and our cohorts were too young.

Limits and strengths of the OTBB3-FUP study: as explained in the design of the study the unexposed cohort was older (36 vs 32 months), most analyses were however conducted with age-adapted scoring. Both cohorts displayed “baseline” characteristics representative of the general neonatal PWS population [23]. The unexposed cohort is small comprising 24 children but they are representative of the natural history of PWS up to 3 years reported in the literature [24–32].

**Conclusions:** these results show for the first time in human that early intranasal OT treatment in neonates and infants with PWS has early and far-reaching positive effects, suggesting that this treatment qualifies as a disease-modifying drug. We anticipate that these results will also have a broad impact on infants with other neurodevelopmental disorders, particularly those with neonatal hypotonia and impaired sucking and swallowing functions.

## Electronic supplementary material

Below is the link to the electronic supplementary material.

Supplementary Material 1

## Data Availability

The data that support the findings of these studies are available from the CHU of Toulouse but restrictions apply to the availability of these data, which were used under license for these studies, and thus are not publicly available. Data are, however, available from the authors upon reasonable request and with permission of the CHU of Toulouse.

## Abbreviations

PWS: Prader-Willi Syndrome
OT: Oxytocin
NOMAS: Neonatal Oral-Motor Scale
VFSS: Videofluoroscopy of Swallowing Study
ADBB: Alarm Distress Baby Scale
CIB: Coding Interactive Behavior scale
AG: Acylated Ghrelin
UAG: Unacylated Ghrelin
rs-fMRI: Resting-state functional Magnetic Resonance Imaging
AE: Adverse Events
VABS-II: Vineland Adaptive Behavior Scales, Second Edition, Third Edition
CMH: Cochran-Mantel-Haenszel test
OR: Odds Ratios
95%CI: 95% Confidence Interval
BSMSS: Barratt Simplified Measure of Social Status
SPS: Social Provision Scale
SDS: Standard Deviation Score
