# Supplementary material for: Oxytocin in infants with Prader-Willi syndrome to improve dysphagia and disease trajectory
Source: Orphanet J Rare Dis. 2026 Feb 4;21:88. doi: 10.1186/s13023-026-04214-8 (PMC12964787; doi:10.1186/s13023-026-04214-8)
Supplement: Supplementary file 1 — Supplementary Material 1 [file 13023_2026_4214_MOESM1_ESM.docx]

Table of contents

[Supplementary Table 1 : VFSS grid and scoring methods 2](file:///Z:\NOUVEAU%20DOSSIER%20RECHERCHE%20SPW\OTBB3\ARTICLE\Article%20Décembre%202025\Supplementary%20data%20revised.edited.docx#_Toc215741874)

[Supplementary MRI methods 4](file:///Z:\NOUVEAU%20DOSSIER%20RECHERCHE%20SPW\OTBB3\ARTICLE\Article%20Décembre%202025\Supplementary%20data%20revised.edited.docx#_Toc215741875)

[Supplementary Table 2. Description of the Treatment-Emergent Adverse Events observed from baseline to week 4 for OT and placebo groups in OTBB3 study reported in at least 2% of patients. 6](file:///Z:\NOUVEAU%20DOSSIER%20RECHERCHE%20SPW\OTBB3\ARTICLE\Article%20Décembre%202025\Supplementary%20data%20revised.edited.docx#_Toc215741876)

[Supplementary Table 3. Prevalence and severity summary of Adverse Events and Treatment-Emergent Adverse Events observed from baseline to week 4 for OT and placebo groups in OTBB3 study. 7](file:///Z:\NOUVEAU%20DOSSIER%20RECHERCHE%20SPW\OTBB3\ARTICLE\Article%20Décembre%202025\Supplementary%20data%20revised.edited.docx#_Toc215741877)

[Supplementary Table 4. Description of Treatment-Emergent Adverse Events observed from Baseline to Week 26 in the 2 groups receiving OT either for 8 Weeks or 4 Weeks in OTBB3 study reported in at least 5% of patients.. 8](file:///Z:\NOUVEAU%20DOSSIER%20RECHERCHE%20SPW\OTBB3\ARTICLE\Article%20Décembre%202025\Supplementary%20data%20revised.edited.docx#_Toc215741878)

[Supplementary Table 5. Overall summary of Adverse Events prevalence and severity observed from baseline to week 26 in the 2 groups receiving OT either for 8 weeks or 4 weeks in OTBB3 study. 9](file:///Z:\NOUVEAU%20DOSSIER%20RECHERCHE%20SPW\OTBB3\ARTICLE\Article%20Décembre%202025\Supplementary%20data%20revised.edited.docx#_Toc215741879)

[OTBB3 Study Personnel List by centers 10](file:///Z:\NOUVEAU%20DOSSIER%20RECHERCHE%20SPW\OTBB3\ARTICLE\Article%20Décembre%202025\Supplementary%20data%20revised.edited.docx#_Toc215741880)

[OTBB3-FUP Study Personnel List by centers 12](file:///Z:\NOUVEAU%20DOSSIER%20RECHERCHE%20SPW\OTBB3\ARTICLE\Article%20Décembre%202025\Supplementary%20data%20revised.edited.docx#_Toc215741881)

# Supplementary Table 1 : VFSS grid and scoring methods

| **Domain** | **Component** | **Observation** | **Results** |
| --- | --- | --- | --- |
| **Oral phase** | Lip closure | Continence | 🞏 Yes *(1pt)* 🞏 No *(2 pts)* |
|  | Mandibular movements | Stability | 🞏 Yes *(1pt)* 🞏 No *(2 pts)* |
|  | Lingual movements | Number of sucking movements per swallowing | 🞏 1 to 2 *(1pt)*  🞏 3  *(2pts)*  🞏 4  *(3pts)*  🞏 > 4 *(4 pts)* |
|  | Coordination of movements | Sucking regular rhythm | 🞏Yes *(1pt)* 🞏 No *(2 pts)* |
| **Pharyngeal swallow initiation and synchronization** | Pharyngeal swallow initiation | Bolus location | 🞏 1/3 posterior of the tongue *(1pt)*  🞏 Vallecula *(2 pts)*  🞏 Pyriform sinus *(3 pts)* |
|  | Swallowing-breathing synchronization | Spontaneous pause | 🞏Infrequent *(1pt)*  🞏 Frequent *(2 pts)*  🞏Very frequent *(3 pts)* |
| **Pharyngeal phase and airway protection** | Velopharyngeal continence | Nasopharyngeal reflux | 🞏 Yes *(2pts)* 🞏 No *(1pt)* |
|  | Pharyngeal propulsion | Presence of residue at:  Vallecula  Pyriform sinus  Pharyngeal wall | 🞏 Yes *(2pts)* 🞏No *(1pt)*  🞏 Yes *(2pts)* 🞏 No *(1pt)*  🞏 Yes *(2pts)* 🞏 No *(1pt)* |
|  | Airway protection | Penetration and aspiration | 🞏 No *(1pt)*  🞏 Expulsed penetration *(2 pts)*  🞏 Non-expulsed penetration *(3 pts)*  🞏 Expulsed aspiration *(4 pts)*  🞏 Non- expulsed aspiration *(5 pts)* |

The VFSS was performed in all centers using the same procedures; all the bottles were made identically (1/3 Barium Sulfate (Contrast product Micropaque®) + 2/3 water).

The scoring grid was used in the previous study (Tauber et al, Pediatrics; 2017:139) to quantify the severity of the swallowing dysfunction in PWS infant.

All normal evaluations are scored 1 and abnormal assessment may be scored 2 to 5. The total score is calculated by adding all points. The minimum score is 11 and the maximum score is 29.

Airway protection: the scoring method for airways protection is based on the Penetration Aspiration Scale (PAS) which was firstly published for VFSS in adults and children above 4 years by Rosenbek JC et al in 1996. The 8 items to evaluate the severity were simplified to 4 by our speech and language expert for infants:

- Expulsed penetration: contrast enters the airway and remains above or contacts the vocal cords – no residue remains after swallowing

- Non expulsed penetration: contrast enters the airway and remains above or contacts the vocal cords –residue remains after swallowing.

- Expulsed aspiration: contrast enters the airway below the vocal cords – no residue remains after swallowing.

- Non expulsed aspiration: contrast enters the airway below the vocal cords –residue -remains after swallowing.

Reference Rosenbek JC, Robbins JA, Roecker EB, et al. A penetration-aspiration scale. Dysphagia 1996;11:93– 8.

# Supplementary MRI methods

Resting-State Functional MRI (Rs-fMRI) were performed in French investigational centers only. Patients were imaged at baseline and at week 4 in conditions of natural rest. The duration of an MRI exam is about 30 minutes (15 minutes set-up and 15 minutes for the exam). Analyses were performed in a centralized manner at Toulouse NeuroImaging Center, UMR1214.

Resting-state fMRI data analysis is described below.

**rs-fMRI preprocessing**

The data were analyzed using the Conn toolbox (Version 21.a), implemented in MATLAB. The preprocessing pipeline of the functional images included: functional realignment and unwarp, slice-timing correction, outlier identification, normalization to the template, and smoothing with a Gaussian kernel of 8 mm. We defined a specific template for this study using T13D of each participant by Diffeomorphic Anatomical Registration Through Exponentiated Lie Algebra DARTEL (toolbox of SPM12). This step created a scrubbing covariate (containing the potential outliers scans for each participant) and a realignment covariate (containing the six head motion parameters). Then, the six head motion parameters plus their associated first-order derivatives, the identified outliers scans, white matter and cerebrospinal fluid signals and the effect of rest were removed by means of the CompCor method. The resulting preprocessed images were band-pass filtered (0.008 Hz–0.25 Hz) to remove physiological high- and low-frequency noise (e.g., cardiac and respiratory fluctuations).

We calculated five different indexes to measure brain connectivity: LocalCorr, GlobalCorr, OFseed, fALFF, ICA-DMN. Figure 3 shows the analyses done with the two later indexes : fALFF, ICA-DMN, which are described below.

**Fractional Amplitude of Low-Frequency Fluctuations (fALFF)**

Fractional Amplitude of Low-Frequency Fluctuations measures the relative contribution of spontaneous low-frequency oscillations within a specific frequency band to the whole detectable frequency range. Fractional Amplitude of Low-Frequency Fluctuations is less prone to noise compared to Amplitude of Low-Frequency Fluctuations (ALFF), which is useful to identify specific local brain areas with abnormal BOLD activity. It is calculated as a relative measure of BOLD signal power within the frequency band compared to that over the entire frequency spectrum. It represents the ratio of root mean square of BOLD signal at each individual voxel after vs. before low- or band- pass filtering (Zou et al. 2008).

**Independent Component Analysis : Default mode Network (ICA-DMN)**

ICA maps represent a measure of different networks expression and connectivity at each voxel. We used GIFT to select the components spatially close to Default Mode Network (DMN) for each subject (using Group ICA fMRI Toolbox [GIFT], http:// mialab. mrn. org/. software/ gift/ , Version .3.0c). Using this approach, we calculated a DMN map for each subject where the value in each voxel is a z score measure of the pixel connectivity to the network.

**Statistical analysis**

The statistical threshold for second level analysis i.e. OT/Placebo comparison or clinical correlations was set at : cluster threshold p<.05 cluster-size p-FDR corrected and voxel threshold p<.001 p-uncorrected. This threshold is the default threshold of CONN software and a classical one in fMRI. In fig 3 we reported regions that survived to a more permissive threshold: cluster threshold p<.05 cluster-size p-uncorrected and voxel threshold p<.001 p-uncorrected and cluster extend=50 voxels.

**References:**

Zou, Q. H., Zhu, C. Z., Yang, Y., Zuo, X. N., Long, X. Y., Cao, Q. J., ... & Zang, Y. F. (2008). An improved approach to detection of amplitude of low-frequency fluctuation (ALFF) for resting-state fMRI: fractional ALFF. *Journal of neuroscience methods*, *172*(1), 137-141.

# Supplementary Table 2. Description of the Treatment-Emergent Adverse Events observed from baseline to week 4 for OT and placebo groups in OTBB3 study reported in at least 2% of patients.

| **Preferred Term** | **Number of patients in OT group (N = 26)** | **Number of patients in Placebo group (N = 26)** | **Total (N = 52)** |
| --- | --- | --- | --- |
| Fatigue | 1 (3.8%) | 3 (11.5%) | 4 (7.7%) |
| Nasopharyngitis | 3 (11.5%) | 1 (3.8%) | 4 (7.7%) |
| Pyrexia | 1 (3.8%) | 3 (11.5%) | 4 (7.7%) |
| Constipation | 2 (7.7%) | 1 (3.8%) | 3 (5.8%) |
| Hypothyroidism | 2 (7.7%) | 1 (3.8%) | 3 (5.8%) |
| Bronchiolitis |  | 2 (7.7%) | 2 (3.8%) |
| Candida infection | 1 (3.8%) | 1 (3.8%) | 2 (3.8%) |
| Decreased appetite |  | 2 (7.7%) | 2 (3.8%) |
| Eczema | 1 (3.8%) | 1 (3.8%) | 2 (3.8%) |
| Hyperkalaemia | 1 (3.8%) | 1 (3.8%) | 2 (3.8%) |
| Hypotonia | 1 (3.8%) | 1 (3.8%) | 2 (3.8%) |
| Rhinitis |  | 2 (7.7%) | 2 (3.8%) |
| Total | 13 | 19 | 32 |

# Supplementary Table 3. Prevalence and severity summary of Adverse Events and Treatment-Emergent Adverse Events observed from baseline to week 4 for OT and placebo groups in OTBB3 study.

|  | **Part 1**  **OT**  **(N=26)** | **Part 1**  **Placebo**  **(N=26)** | **Total**  **(N=52)** |
| --- | --- | --- | --- |
| Patients with at least one AE | 18 (69.2%) [27] | 15 (57.7%) [37] | 33 (63.5%) [64] |
| Patients with at least one TEAE | 16 (61.5%) [21] | 15 (57.7%) [32] | 31 (59.6%) [53] |
| Patients with at least one Treatment-related TEAE | 1 (3.8%) [1] | 1 (3.8%) [2] | 2 (3.8%) [3] |
| Patients with at least one TEAE by greatest severity [1]  Grade 1 (Mild)  Grade 2 (Moderate)  Grade 3 (Severe) | 13 (50.0%) [16]  3 (11.5%) [5]  0 (0%) [0] | 11 (42.3%) [24]  4 (15.4%) [7]  0 (0%) [0] | 24 (46.2%) [40]  7 (13.5%) [12]  0 (0%) [0] |
| Patients with at least one serious AE | 2 (7.7%) [2] | 0 (0%) [0] | 2 (3.8%) [2] |
| Patients with at least one serious TEAE | 1 (3.8%) [1] | 0 (0%) [0] | 1 (1.9%) [1] |
| Patients with at least one serious Treatment-Related TEAE | 0 (0%) [0] | 0 (0%) [0] | 0 (0%) [0] |
| Patients with at least one TEAE leading to study drug discontinuation | 0 (0%) [0] | 1 (3.8%) [3] | 1 (1.9%) [3] |
| Patients with at least one AE leading to death | 0 (0%) [0] | 0 (0%) [0] | 0 (0%) [0] |

OT = Oxytocin; Plb = Placebo;

[1] Each patient is counted only once within the greatest reported severity (i.e. each patient is counted in one category only). Events under the patient’s greatest reported severity counted.

AE = Adverse event; E = Number of events; TEAE = Treatment-emergent adverse event.

Not(s): Only AEs/TEAEs occurring from study start to Week 4 are included. Treatment-emergent adverse events are defined as any event that started on or after the first study medication administration or worsening during study treatment. Treatment-related adverse events are defined as any AE assessed by the Investigator as “Definitively related”, “Probably related” or “Potentially related”. TEAEs assessed as “Unlikely related” or “Not related” are defined as not related. TEAE leading to study drug discontinuation includes events leading to temporary discontinuation and events leading to permanent discontinuation from study drug.

Adverse events are coded using MedDRA version 24.1.

The Serious TEAE reported in the OT group was a worsening of alveolar hypoventilation which resolved after for 5 days of hospitalization.

# Supplementary Table 4. Description of Treatment-Emergent Adverse Events observed from Baseline to Week 26 in the 2 groups receiving OT either for 8 Weeks or 4 Weeks in OTBB3 study reported in at least 5% of patients.

| **Preferred Term** | **Number of patients in Group**  **8 Weeks OT (N = 27)** | **Number of patients in Group**  **4 Weeks OT (N = 25)** | **Total (N = 52)** |
| --- | --- | --- | --- |
| Bronchiolitis | 7 (25.9%) | 6 (24.0%) | 13 (25.0%) |
| Pyrexia | 6 (22.2%) | 7 (28.0%) | 13 (25.0%) |
| Nasopharyngitis | 3 (12.0%) | 8 (32.0%) | 11 (21.2%) |
| Hypothyroidism | 2 (7.4%) | 6 (24.0%) | 8 (15.4%) |
| Eczema | 1 (3.7%) | 6 (24.0%) | 7 (13.5%) |
| Rhinitis | 3 (11.1%) | 3 (12.0%) | 6 (11.5%) |
| Anemia | 2 (7.4%) | 3 (12.0%) | 5 (9.6%) |
| Conjunctivitis | 3 (11.1%) | 2 (8.0%) | 5 (9.6%) |
| Diarrhea | 2 (7.4%) | 3 (12.0%) | 5 (9.6%) |
| Ear infection | 2 (7.4%) | 3 (12.0%) | 5 (9.6%) |
| Overdose | 3 (11.1%) | 2 (8.0%) | 5 (9.6%) |
| Constipation | 2 (7.4%) | 2 (8.0%) | 4 (7.7%) |
| Fatigue | 2 (7.4%) | 2 (8.0%) | 4 (7.7%) |
| Oral candidiasis | 2 (7.4%) | 2 (8.0%) | 4 (7.7%) |
| Varicella | 2 (7.4%) | 2 (8.0%) | 4 (7.7%) |
| Decreased appetite | 2 (7.4%) | 1 (4.0%) | 3 (5.8%) |
| Erythema | 2 (7.4%) | 1 (4.0%) | 3 (5.8%) |
| Fungal infection | 2 (7.4%) | 1 (4.0%) | 3 (5.8%) |
| Gastroesophageal reflux disease | 1 (3.7%) | 2 (8.0%) | 3 (5.8%) |
| Pyelonephritis acute | 3 (11.1%) | 0 (0.0%) | 3 (5.8%) |
| Urinary tract infection | 2 (7.4%) | 1 (4.0%) | 3 (5.8%) |
| Total | 54 | 63 | 117 |

# Supplementary Table 5. Overall summary of Adverse Events prevalence and severity observed from baseline to week 26 in the 2 groups receiving OT either for 8 weeks or 4 weeks in OTBB3 study.

|  | **8 weeks**  **OT**  **(N=27)** | **4 weeks**  **OT**  **(N=25)** | **Total**  **(N=52)** |
| --- | --- | --- | --- |
| Patients with at least one AE | 27 (100%) [111] | 24 (96%) [112] | 51 (98.1%) [223] |
| Patients with at least one TEAE | 27 (100%) [107] | 24 (96%) [105] | 51 (98.1%) [212] |
| Patients with at least one TEAE by greatest severity [1]  Grade 1 (Mild)  Grade 2 (Moderate)  Grade 3 (Severe) | 16 (59.3%) [54]  8 (29.6%) [19]  3 (11.1%) [3] | 17 (68.0%) [77]  6 (24.0%) [8]  1 (4.0%) [1] | 33 (63.5%) [131]  14 (26.9%) [27]  4 (7.7%) [4] |
| Patients with at least one Treatement-related TEAE | 4 (14.8%) [7] | 2 (8.0%) [2] | 6 (11.5%) [9] |
| Patients with at least one serious AE | 7 (25.9%) [12] | 6 (24.0%) [9] | 13 (25.0%) [21] |
| Patients with at least one serious TEAE | 7 (25.9%) [12] | 6 (24.0%) [8] | 13 (25.0%) [20] |
| Patients with at least one serious Treatment-Related TEAE | 1 (3.7%) [1] | 0 (0%) [0] | 1 (1.9%) [1] |
| Patients with at least one TEAE leading to study drug discontinuation | 1 (3.7%) [3] | 0 (0%) [0] | 1 (1.9%) [3] |
| Patients with at least one AE leading to death | 0 (0%) [0] | 0 (0%) [0] | 0 (0%) [0] |

OT = Oxytocin; Plb = Placebo;

[1] Each patient is counted only once within the greatest reported severity (i.e. each patient is counted in one category only). Events under the patient’s greatest reported severity counted.

AE = Adverse event; E = Number of events; TEAE = Treatment-emergent adverse event.

Note(s): All AEs/TEAEs occurring during the study are included. Treatment-emergent adverse events are defined as any event that started on or after the first study medication administration or worsening during study treatment. Treatment-related adverse events are defined as any AE assessed by the Investigator as “Definitively related”, “Probably related” or “Potentially related”. TEAEs assessed as “Unlikely related” or “Not related” are defined as not related. TEAE leading to study drug discontinuation includes events leading to temporary discontinuation and events leading to permanent discontinuation from study drug.

Adverse events are coded using MedDRA version 24.1.

The serious treatment related TEAE observed in 1 patient who received 8 weeks of OT was an intracranial hypertension which occurred 2 months after cessation of treatment and resolved after 1 month of hospitalization.

Three severe TEAE were reported in infants who received 8 weeks of OT:

- 1 infant had acute pyelonephritis which occurred 4 months after cessation of treatment and was resolved after 5 days of hospitalization

- 1 had intestinal obstruction 2 months after cessation of treatment which resolved after 40 days of hospitalization; this infant also had intracranial hypertension (see above).

- 1 had bronchiolitis which occurred 2 weeks after cessation of treatment and resolved after 10 days of hospitalization. He never received treatment for esophageal reflux.

One severe TEAE was reported in infant who received 4 weeks of OT; he had bronchiolitis 3 months after cessation of treatment which resolved after 3 weeks of hospitalization. He received treatment for esophageal reflux (esomeprazole) since birth.

# OTBB3 Study Personnel List by centers

COORDINATING INVESTIGATOR: Pr Maithe TAUBER

- France:
  - Toulouse:

Investigators: Dr Gwenaelle DIENE, Pr Maithe TAUBER, Dr Marine DELAGRANGE, Dr Sophie MONIEZ

PWS Reference Centre clinical team: Dr Sophie ÇABAL, Dr Pascale FICHAUX-BOURIN, Mélanie GLATTARD, Christelle NOUAL

PWS Reference Centre research team: Marion VALETTE, Catherine MOLINAS, Cathy BROCHADO, Sandy FAYE, Julie CORTADELLAS

Radiologist: Dr Julie VIAL

MRI unit : Pr Pierre PAYOUX, Hélène GROS, Nathalie VAYSSIERE

- - Paris Necker:

Investigators: Dr Graziella PINTO, Dr Véronique ABADIE, Dr Emeline ROY

Pediatric endocrinology team: Anne-Claire DORSEMANS, Gaëlle MALECOT, Aurélie ROYER

Pediatric Clinical Investigation Centre: Dr Michaela SEMERARO, Solimda SOTOU BERE TCHAO, Anaïs LEON, Linda IMIKIRENE, Carolina PIERRARD, Saphia FAKED

Radiologist: Dr Raphaël LEVY

MRI unit: Pr Nathalie BODDAERT

- - Marseille:

Investigators: Pr Rachel REYNAUD, Dr Delphine BERNOUX

Pediatric Clinical Investigation Centre: Valentine VERDIER, AGUZZI Anthony, ATTOLINI Laurence, Sarah CZIESLA

Radiologist: PrPhilippe PETIT

MRI unit: Pr Nadine GIRARD

- - Lille:

Investigator: Dr Iva Gueorguieva

Pediatric Clinical Investigation Centre : Dr Florence FLAMEIN, Cécile EVRARD, Laurence MORTREUX, Amina ZIOUCHE, Emilie BOUVY, Laurent BEGHIN

Radiologists: Dr Héloïse LERISSON, Dr Céline TILLAUX

MRI unit: Dr Gustavo SOTO ARES

- - Lyon:

Investigators: Pr Marc NICOLINO, Dr Emilie DOYE, Dr Aurélie PORTEFAIX

Pediatric Clinical Investigation Centre : Segolene GAILLARD, Tiphaine GINHOUX, Sandrine MARDIROSSIAN, Iris LEMERCIER, Delphine DAURADE-GROSJEAN

Radiology: Adeline MANSUY, Morgane BOUIN, Claire GUICHARD

MRI unit: Franck LAMBERTON

- Belgium: Brussels

Investigators: Pr Véronique BEAULOYE, Pr Elin MALEK ABRAHIMIANS

Pediatric endocrinology team: Dr Sophie SIMMANN, Nathalie JODOGNE,

Pediatric Clinical Investigation Centre : Vanessa JACOBS, Julia VERSAVAU, Sandrine LAMBERT

Radiologist : Dr Perrine Triqueneaux

- GERMANY: Essen

Investigators: Dr Cordula KIEVERT, Dr Martin MUNTEANU, Dr Raphael HIRTZ

Pediatric endocrinology team: Wiebke DIESNER

# OTBB3-FUP Study Personnel List by centers

COORDINATING INVESTIGATOR: Pr Maithe TAUBER

- Toulouse:

Investigators: Dr Gwenaelle DIENE, Pr Maithe TAUBER, Dr Sophie ÇABAL, Dr Alice CLERC

PWS Reference Centre clinical team: Dr Pascale FICHAUX-BOURIN, Mélanie GLATTARD, Christelle NOUAL

PWS Reference Centre research team: Marion VALETTE, Catherine MOLINAS, Cathy BROCHADO, Sandy FAYE, Julie CORTADELLAS

Radiologist: Dr Julie VIAL

- Paris Necker:

Investigators: Dr Graziella PINTO, Dr Romain BERTHAUD

Pediatric endocrinology team: Anne-Claire DORSEMANS

Pediatric Clinical Investigation Centre: Dr Michaela SEMERARO, Aude BOYER, Saphia FAKED, Sofia BOUADJIL, Laure GALLOIS

Radiologists: Pr Nathalie BODDAERT, Dr Elisa ZANELLI

- Marseille:

Investigators: Pr Rachel REYNAUD, Dr Delphine BERNOUX, Dr Julia VERGIER, Dr Alexia DABADIE

Pediatric endocrinology team : Cécile THOMAS, Mathilde DE SAINT CHERON

Pediatric Clinical Investigation Centre : Graziela PREDA CAROSINI, Valentine VERDIER, Sarah CZIESLA, AGUZZI Anthony, ATTOLINI Laurence

Radiology unit : Dr Harmony PICO, Dr Nathalie COLAVOLPE, Pr Philippe PETIT, Séverine SAURIER

- Lille:

Investigator: Dr Iva Gueorguieva, Dr Christine LEFEVRE

Pediatric endocrinology team: Samatha MEYER, Nathan STORME, Hugues BACHELART

Pediatric Clinical Investigation Centre : Dr Florence FLAMEIN, Cécile EVRARD, Laurence MORTREUX, Amina ZIOUCHE, Emilie BOUVY, Michael HISBERGUES

Radiologist: Dr Héloïse LERISSON

- Lyon:

Investigators: Pr Marc Nicolino, Dr Aurélie PORTEFAIX

Pediatric endocrinology team: Tiphaine FRANCOIS, Logan BARS, Karima BELHASSEN, Caroline VILLALBA

Pediatric Clinical Investigation Centre : Iris LEMERCIER, Leslie REYROLLE

Radiologists: Dr Isabelle CANTERINO, Dr Loic VIREMOUNEIX

- Nice:

Investigator: Dr Elsa HAINE, Dr Cécile DARNAUD

Pediatric endocrinology team: Carole DIDIER, Ana GIORDANO, Sonanda BAILLEUX

Pediatric Clinical Investigation Centre : Luc FROISSANT, Aline JOULIE

Radiologist: Dr Marco ALBERTARIO

- Grenoble:

Investigators: Dr Anne SPITERI, Dr Cyril RUELLO

Pediatric clinical team: Karine GUICHARDET, Ludovic GIRAUD

Pediatric Clinical Investigation Centre: Noëlle BROSILLE, Caroline TOURNEGROS

Radiologist: Dr Marco ALBERTARIO

- Dijon:

Investigators: Dr Marie BOURNEZ, Dr Candace BEN SIGNOR

Pediatric clinical team : Elisa CACAUD

Pediatric Clinical Investigation Centre : Mélodie SOTO, Barbara LEXTREYT, Julie MARÉCHAL, Thi Thu CREUSVAUX-NGUYEN

Radiologist: Dr Adélaïde REGA

- Nantes:

Investigators: Dr Sabine BARON, Dr Emmanuelle CALDAGUES

Pediatric clinical team: Dr Laetitia ROBARD, Dr Julie BOYER, Dr Batoul SAMARJI, Elodie LEMARDIE

Pediatric Clinical Investigation Centre : Solène EGRON, Maud GUILLET, Alix LAURENT, Anne CHAUVIRE-DROUARD, Thierry BOMPOIL

Radiologist: Dr Pascale GUILLOT

- Rennes:

Investigators: Dr Marie-Beatrice SAADE

Pediatric clinical team: Fanny TESSIER

Pediatric Clinical Investigation Centre: Elisabeth POIREL

Radiologist: Dr Laetitia ROBARD, Dr Valérie CHARON

- Rouen:

Investigators: Dr Mireille CASTANET, Dr Claire GAYET

Pediatric clinical team : Camille WEITZ

Pediatric Clinical Investigation Centre : Solenn COCHET, Carine CHOUBRAC, Alexandra BOUGEARD, Melody DE JESUS

Radiologists: Dr Ioana VASIES, Dr Nicolas SENS
